# NKCC1 up-regulation contributes to early post-traumatic seizures and increased post-traumatic seizure susceptibility

**DOI:** 10.1007/s00429-016-1292-z

**Published:** 2016-09-01

**Authors:** Fushun Wang, Xiaowei Wang, Lee A. Shapiro, Maria L. Cotrina, Weimin Liu, Ernest W. Wang, Simeng Gu, Wei Wang, Xiaosheng He, Maiken Nedergaard, Jason H. Huang

**Affiliations:** 1 Nanjing University of Chinese Medicine, Nanjing, 210023 China; 2 Division of Glial Disease and Therapeutics, Center for Translational Neuromedicine, University of Rochester, Rochester, NY 14642 USA; 3 Neuroscience Graduate Program, University of Rochester, Rochester, NY 14642 USA; 4 Department of Surgery, Texas A&M University Health Science Center, College of Medicine, Temple, TX 76504 USA; 5 Department of Neurosurgery, Neuroscience Institute, Baylor Scott and White Health, Central Division, Temple, TX 76508 USA; 6 Department of Neurosurgery, Xijing Hospital, 4th Military Medical University, Xi’an, China

**Keywords:** Traumatic brain injury, In vivo electrophysiology, TGF, Bumetanide

## Abstract

Traumatic brain injury (TBI) is not only a leading cause for morbidity and mortality in young adults (Bruns and Hauser, Epilepsia 44(Suppl 10):210, 2003), but also a leading cause of seizures. Understanding the seizure-inducing mechanisms of TBI is of the utmost importance, because these seizures are often resistant to traditional first- and second-line anti-seizure treatments. The early post-traumatic seizures, in turn, are a contributing factor to ongoing neuropathology, and it is critically important to control these seizures. Many of the available anti-seizure drugs target gamma-aminobutyric acid (GABA_A_) receptors. The inhibitory activity of GABA_A_ receptor activation depends on low intracellular Cl^−^, which is achieved by the opposing regulation of Na^+^–K^+^–Cl^−^ cotransporter 1 (NKCC1) and K^+^–Cl^−^–cotransporter 2 (KCC2). Up-regulation of NKCC1 in neurons has been shown to be involved in neonatal seizures and in ammonia toxicity-induced seizures. Here, we report that TBI-induced up-regulation of NKCC1 and increased intracellular Cl^−^ concentration. Genetic deletion of NKCC1 or pharmacological inhibition of NKCC1 with bumetanide suppresses TBI-induced seizures. TGFβ expression was also increased after TBI and competitive antagonism of TGFβ reduced NKKC1 expression, ameliorated reactive astrocytosis, and inhibited seizures. Thus, TGFβ might be an important pathway involved in NKCC1 up-regulation after TBI. Our findings identify neuronal up-regulation of NKCC1 and its mediation by TGFβ, as a potential and important mechanism in the early post-traumatic seizures, and demonstrate the therapeutic potential of blocking this pathway.

**Electronic supplementary material:**

The online version of this article (doi:10.1007/s00429-016-1292-z) contains supplementary material, which is available to authorized users.

## Introduction

Traumatic brain injury (TBI) is a leading cause for death and disability (Huang 2013; Ren et al. 2013; Wang et al. 2013; Algattas and Huang 2014), annually affecting over one and a half million people in the United States (Arndt et al. 2013; Huang 2013). The early post-traumatic seizures are one of the most common complications resulting from TBI, the incidence of which ranges from 4.4 to 53 % (Frey 2003; Oberheim et al. 2008). The early post-traumatic seizures can be defined as seizures that occur within 1 week of a brain injury. Approximately 25 % of patients who experience an early post-traumatic seizure will have another seizure months or years later (Liesemer et al. 2011). In general, the early post-traumatic seizures are correlated with the severity of head injury, and they may also be related to age (Chiaretti et al. 2000; Petridis et al. 2012). Moreover, as many as 26–52 % of TBI patients suffer from non-convulsive or subclinical seizures, which may escape clinical observations and can only be detected with electroencephalogram (EEG) (Saengpattrachai et al. 2006; Rodgers et al. 2015). Therefore, the true incidence of the early post-traumatic seizures may be significantly under-reported.

The early post-traumatic seizures may indicate ongoing cerebral injury processes, such as: inflammation, neurodegeneration, aberrant plasticity, hemorrhage, and edema. In addition, the early post-traumatic seizures can also cause secondary brain injury by increasing metabolic requirements and cerebral blood flow, elevating intracranial pressure leading to cerebral hypoxia/ischemia, exacerbating indiscriminate neurotransmitter release, and elevating brain temperature to a harmful level (Tyor et al. 2002; Vespa et al. 2007; Algattas and Huang 2014). Therefore, it is critically important to control the early post-traumatic seizures in TBI patients. However, the mechanisms of the early post-traumatic seizures remain elusive, and the treatments are limited to traditional anticonvulsant and anti-epileptic drugs. This is potentially problematic, because these drugs are designed to treat epileptic seizures, and the early post-traumatic seizures are distinctly different from epileptic seizures. As such, this treatment strategy may not always provide adequate control over the early post-traumatic seizures or prevent further brain injury (Beghi 2003; Arndt et al. 2013).

Prospective randomized trials have shown that prophylactic anticonvulsants may prevent the occurrence of the early seizures in TBI patients (Temkin et al. 1990). Many of these drugs block seizures by enhancing inhibitory GABA_A_ receptor activity in the brain. The inhibitory activity of GABA_A_ receptor activation is dependent on low intracellular Cl^−^. The modulation of intracellular Cl^−^ occurs by opposing activity of Na^+^–K^+^–2Cl^−^ cotransporter (NKCC1) and K^+^–Cl^−^ cotransporter (KCC2) in neurons (Fu et al. 2015; Modol et al. 2015). Up-regulation of NKCC1 will lead to increased intracellular Cl^−^, which, in turn, renders GABAergic input less inhibitory and more seizure prone. We have recently reported that NKCC1 function increased under ammonia toxicity and might be a key mechanism for ammonia-induced seizures (Rangroo Thrane et al. 2013). The fact that NKCC1 and thus Cl^−^ homeostasis are altered after TBI (Kahle et al. 2008) suggests that this may be one mechanism for neuronal hyper-excitability in the early post-traumatic seizures. Therefore, in this study, we investigated the role of NKCC1, and associated mechanisms, in seizure activity after TBI.

## Materials and methods

### Mice and TBI

Male C57bc/6j mice were obtained from Jackson Laboratories. Mice at 6–8 weeks old were subjected to a single, closed-head, unilateral cortical injury, as previously described (Petraglia et al. 2014a, b). Briefly, awake un-anesthetized mice were restrained in DecapiCones (Braintree Scientific, Inc.) for 10–20 s during the injury to achieve head restriction relative to the body (6 mm in diameter and 2 mm in thickness) covering the target area of injury (left parietal, center of the disc 5–7 mm from sagittal suture) by securing it to the mouse head to prevent skull fracture. The injury device includes a pneumatic-controlled impactor (Pittsburgh Precision Instruments, Inc.) with a rubber tip attached to a stainless steel piston used to create the impact, and a foam platform, on which the animals were positioned. Before producing the injury, the impactor tip was aligned with the metal disc to ensure the consistency of the injury. Brain injury was delivered by the injury device mounted at 15° from the perpendicular to create a head displacement of 1 cm at a velocity of 7.14 m/s during 100 ms period. Sham mice received identical treatment, but did not receive the TBI.

Slc12a2^−/−^ mice were generated by Delpire et al. (1999), and breeding and genotyping were carried out as per their descriptions (Delpire et al. 1999). As above, male mice, at 6–8 weeks old, were used. These mice (*N* = 26), and the wild-type littermates (*N* = 28) were bred in our facility and used in experiments comparing wild type with Slc12a2^−/−^ mice. All animal experiments were approved by the Animal Care and Use Committee of the University of Rochester.

### Behavioral seizure characterization

Mice were video-taped for 4 h after the injury to characterize their early post-traumatic seizure behavior. Two to three random 5 min periods per hour of recordings were blindly analyzed (a total ten periods were analyzed per mouse). A scoring system adapted from Racine scale was used to quantify the seizure-related behaviors in the immediate phase: 1, immobility; 2, head nodding, stiff tail; 3, isolated myoclonic jerks; 4, clonic seizure; 5, tonic–clonic seizure; 6, convulsion with jumping; and 7, convulsion culminating in death.

### Animal preparation for awake in vivo recordings

Mouse preparations were modifications of the previous protocols (Dombeck et al. 2007; Thrane et al. 2012). EEG recordings were carried out with a tungsten wire implanted under the skull and touching the dura 1 day before the TBI, as previously reported (Petraglia et al. 2014a, b). These recordings were performed for half an hour sessions, randomly selected at 0, 3, or 24 h after TBI. EEG signals were externally filtered at 60 Hz (Filter Butterworth Model by Encore, Axopatch 200B by Axon Instruments), bandpass filtered at 1–100 Hz, and digitized (Digidata 1440A by Axon Instruments). Recordings were analyzed offline using pClamp 10.2. The total power at different frequencies was added together to get normalized power. Myoclonic seizures were defined on the EEG/ECoG as single or multiple 3–9 Hz polyspike and wave discharges of 0.2–2 s duration associated with myoclonic jerks determined by video recording or direct observation.

### In vitro electrophysiology

Unless otherwise noted, 6-week-old C57BL/6 (Jackson Laboratories) were used for the preparation of cortical brain slices as previously described (Wang et al. 2012a). The mice were anesthetized with isoflurane in a closed chamber (1.5 %) and decapitated. The brains were rapidly removed and immersed in ice-cold cutting solution that contained 230 mM sucrose, 2.5 mM KCl, 0.5 mM CaCl_2_, 10 mM MgCl_2_, 26 mM NaHCO_3_, 1.25 mM NaH_2_PO_4_, and 10 mM glucose (pH 7.2–7.4). Coronal slices (400 mm) were cut with a vibratome (Vibratome Company) and transferred to oxygenated aCSF that contained 126 mM NaCl, 4 mM KCl, 2 mM CaCl_2_, 1 mM MgCl_2_, 26 mM NaHCO_3_, 1.25 mM NaH_2_PO_4_, and 10 mM glucose (pH 7.2–7.4, osmolarity = 310 mosM). Slices were incubated in aCSF for 1–5 h at room temperature before recording. Experiments were performed at room temperature (21–23 °C). During the recordings, the slices were placed in a perfusion chamber and superfused with aCSF gassed with 5 % CO_2_ and 95 % O_2_ at room temperature. Cells were visualized with a 40 × water-immersion objective and differential inference contrast (DIC) optics (BX51 upright microscope, Olympus Optical). Patch electrodes were fabricated from filament thin-wall glass (World Precision Instruments) on a vertical puller; the resistance of the pipette was about 6–9 MΩ with intracellular pipette solution added. GABA currents were recorded under voltage clamp with an Axopatch MultiClamp 700B amplifier (Axon Instruments). The pipette solution contained 140 mM K-gluconate, 5 mM Na-phosphocreatine, 2 mM MgCl_2_, 10 mM Hepes, 4 mM Mg-ATP, and 0.3 mM Na-GTP (pH adjusted to 7.2 with KOH). In addition, gramicidin was diluted in methanol to make a stock solution at 10 mg/ml first and then made to the final 50–100 µg/ml in the pipette solution. The junction potential between the patch pipette and the bath solution was zeroed before forming a gigaseal. Patches with seal resistances of less than 1 GΩ were rejected. Data were low-pass filtered at 2 kHz and digitized at 10 kHz with a Digidata 1440 interface controlled by the pClamp Software (Molecular Devices).

### Second-hit PTZ seizure challenge

To assess post-traumatic seizure susceptibility, we administered PTZ (10 mg/kg; i.p.) at 3 days after TBI. For the first series of experiments, we only used those mice that did not experience any early post- traumatic seizures (*N* = 5, slc12a2^+/+^ and *N* = 5 slc12a2^−/−^). The latency and duration of seizures were assessed with EEG recording. We also used PTZ challenge to determine if bumetanide (Sigma Aldrich; 30 mg/kg, i.p., dissolved in DMSO, then diluted with 0.9 % saline) would protect against post-traumatic seizure susceptibility. Mice were subjected to TBI, and 3 days after, bumetanide was injected i.p., 30 min before application of PTZ (10 mg/kg, i.p.) to allow bumetanide to pass through the blood–brain barrier (Tollner et al. 2014). Behavioral and electrophysiological analysis of seizures was performed as above.

A third set of PTZ seizure challenge experiments were performed to assess the effects of the TGF-β blocker LY-364947 on post-traumatic seizure susceptibility. For these studies, mice were subjected to TBI. LY-364947 (TOCRIS, 10 mg/kg, i.p.) was injected once daily for 3 days, with the first injection occurring immediately after the TBI. At 3 days after TBI, PTZ (10 mg/kg, i.p.) was injected and the analysis of seizures was performed as above.

### Immunohistochemistry

Mice were anesthetized under ketamine (100 mg/ml), xylazine (20 mg/ml), and cocktail (0.1 ml per 10 g of body weight), and perfused transcardially with 4 % paraformaldehyde, and the brains were post-fixed overnight. Serial 20 µm coronal cryostat sections were cut after overnight cryoprotection in 15 % and then 30 % sucrose. Sections were incubated with single or combination of following primary antibodies overnight at 4 °C: rabbit anti-NKCC1 primary antibody (1:500, ABCam), mouse anti-KCC2 (1:500, ABCam), mouse anti-TGF beta 2 (1:1000, ABCam), mouse anti-NeuN (1:1000, Millipore), and rabbit anti-GFAP (1:1000 Sigma). The primary antibody detection was followed by incubation with secondary fluorescent antibodies. DAPI (Vector) was used for mounting.

### Cell counts

Images were taken with a 20× lens in a BX53 Olympus system microscope attached to a DP72 Olympus digital camera, or with an Olympus laser-scanning confocal microscope (IX-82). The brightness and contrast of all images were adjusted using Adobe Photoshop (V. CS5). No other adjustments were made to any of the images. We captured 3–5 images per slice, in the areas between 1.2 and −1.8 from bregma, and assured that the regions of interest were sampled equally. Quantification of NKCC1- and KCC2-expressing neurons was performed using computer-assisted analysis (ImageJ software V. 2.0.0-beta-7.8). To randomly select regions of interest for analysis, a 2500 µm box (50 × 50 µm) was randomly placed bi-laterally, over the images-containing somatosensory and motor cortex. Pooled data from both hemispheres were used in the final analysis. For each time point, and for sham, we analyzed four mice per group. We placed three random boxes per slice and analyzed 3–5 images per slice, per mouse, per group.

### Western blot analysis

Western blot analysis for NKCC1 expression change in the posttraumatic cortex was done as previously described (Tong et al. 2014). Briefly, mice were anesthetized and perfused with PBS. Cortex was dissected from the rest of brain tissue in ice-cold PBS using a fine-straight and a fine-angled dissecting forceps. The brain tissue was then lysed on ice in RIPA lysis buffer (150 mM sodium chloride, 1.0 % Triton X-100, 0.5 % sodium deoxycholate, 0.1 % SDS, 50 mM Tris, and pH 8.0) with protease inhibitor (Roche). Protein concentration was determined by Pierce BCA Protein Assay Kit (Thermo scientific). Samples each containing 100 µg of total protein were resolved on SDS-PAGE (10 % gel) in Laemmli Sample Buffer (Bio rad) and transferred to PVDF membranes (Immobilon-FL, Millipore) in transfer buffer for 1 h at room temperature. Membranes were blocked with Odyssey Blocking Buffer at room temperature for 2 h, then incubate with anti-NKCC1 primary antibody (1:500, AbCam), anti-KCC2 primary antibody (1:1000, ABCam), and anti-β-actin at 4 °C overnight. Blots were then probed with florescent secondary antibodies and imaged using the LI-COR Odyssey system. Band intensity was quantified using the LI-COR Odyssey software. Relative protein expression levels were calculated by normalizing the band intensity of the target protein to its respective β-actin loading control.

### Quantitative PCR

Mice were anesthetized under ketamine (100 mg/ml), xylazine (20 mg/ml), and cocktail (0.1 ml per 10 g of body weight), and perfused transcardially with PBS. Cortex and hippocampus were carefully dissected from each hemisphere in ice-cold PBS and immediately transferred to dry ice. Total RNA were prepared using TRIzol^®^ reagent (life technologies) and treated with Turbo DNA-free™ (Life Technologies) to remove DNA contamination. RNA samples were reverse transcribed by TaqMan Reverse Transcription Reagents (life technologies). Relative transcription level of NKCC1 was detected by TaqMan probe (life technologies, Mm01265951_m1) on an ABI Prism 7000 apparatus (Applied Biosystems) in three independent experiments. GAPDH (life technologies, CAT No. Mm99999915_g1) was used as an internal control. The comparative threshold (Δ*C*_t_) method was used and results were converted to fold of expression relative to sham group.

### Densitometry

Images captured using the Olympus IX82 microscope were analyzed using Image J (1.48V), as previously described. Briefly, a standard cutoff is used, such that pixels above a pre-determined intensity cutoff are counted by the program, and those that fall below the threshold are omitted from counting. Prior to thresholding the image to detect NKCC-labeled structures, a background image was created using median filtering. This background image was subtracted from the densitometric analysis. We analyzed 4–6 sections per animal and 3–4 animals per group. Slides were coded and images were captured from corresponding regions of the hippocampus. The analysis was then performed on the coded images, after which the codes were broken and the data analysis performed.

### Statistical analysis

All analysis was performed using the SPSS 19 software (IBM), and all tests were two tailed, where significance was achieved at *α* = 0.05 level. Where appropriate, either an unpaired *t* test (≤2 variables) or one-way ANOVA (>2 variables) was used for independent samples. Where *N* < 10 or the data were non-normally distributed, we employed non-parametric tests, including Mann–Whitney *U* (≤2 variables) or Kruskall–Wallis (>2 variables), that were used for independent samples, and Wilcoxon signed ranks test for paired samples. For all graphs, error bars represent ±standard error of the mean (SEM).

## Results

### Increased NKCC1 and decreased KCC2 expression after TBI

**Fig. 1:**
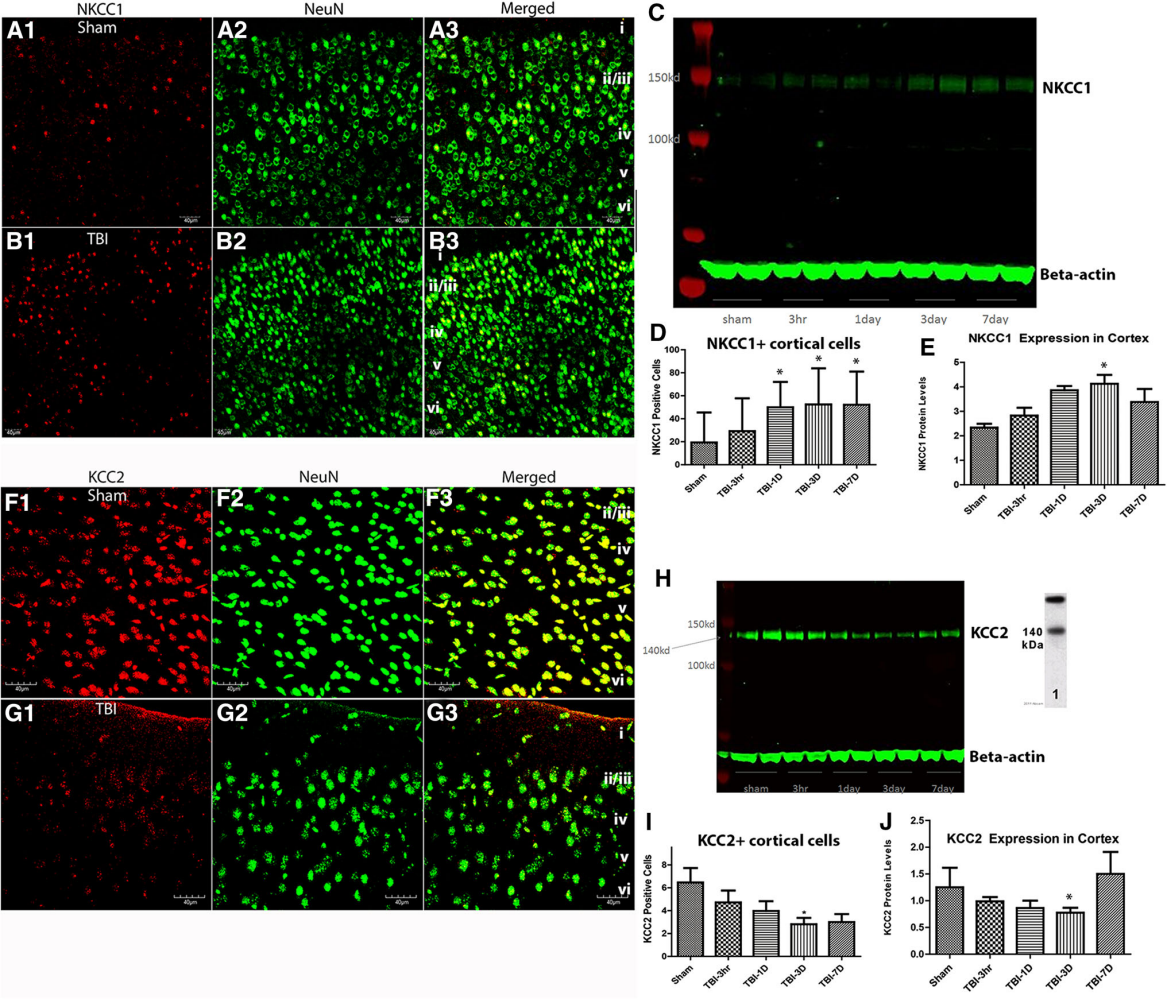
Increased NKCC1 and decreased KCC2 after TBI. **a**, **b** Immunohistochemical staining of NKCC1 (**a1**, **b1**), NeuN (**a2**, **b2**), and the merged image (**a3**, **b3**) are illustrated to show neuronal NKCC1 in layers *i*–*vi* of cortex, at 3 days following sham (**a1**–**a3**) and TBI (**b1**–**b3**) mice. In **c**, an example of a western blot assessing NKCC1 in sham and TBI mice at 3 h, 1, 3, and 7 days post-FPI. Quantitative analysis of NeuN^+^/NKCC1^+^ cells (**d**) revealed a significant increase after by 1 day post-TBI and persisting until at least 7 days after TBI. The counts represent number of NKCC1^+^/NeuN^+^ cells per 25,000 µm^2^. Consistent with this finding, western blot analysis (**e**) of NKCC1 in cortical tissue revealed a significant increase (*F* (4,15) = 4.868, *P* < 0.05, one-way ANOVA) at 3 days post-TBI. It is pertinent to note that we also present qPCR analysis in both cortex and hippocampus, and immunohistochemistry in cortex, showing that the elevation of NKCC1 is bilateral (see Supplementary Figs. 1 and 2). In **f**–**g**, KCC2 and NeuN double-labeling is shown to depict a decrease in KCC2 following TBI (**g1**–**g3**) compared with shams (**f1**–**f3**). **h** Western blot assessing KCC2 in sham and TBI mice at 3 h, 1, 3, and 7 days post-FPI. Quantitative analysis of the NeuN^+^/KCC2^+^ cells (**i**) demonstrated that a significant decrease by 3 days after TBI. The counts represent number of KCC2^+^/NeuN^+^ cells per 25,000 µm^2^ of primary motor and somatosensory cortex. Western blot analysis (**j**) revealed that protein levels of KCC2 were significantly decreased (*F* (4,15) = 1.716, *P* < 0.05) in cortex by 3 days after TBI

We used western blot, and immuno-fluorescent labeling to characterize the expression of NKCC1 and KCC2 after TBI and compared the results with that from sham controls. Quantitative analysis of NKCC1^+^ neurons in cortex revealed a significant increase by 1 day after TBI (Fig. 1a–e). The results from the analysis of western blots (Fig. 1c, e) show a significant increase in NKCC1 in neocortex (*F* (4,15) = 1.7164; *P* < 0.05 at 3 days post-TBI). Quantification of KCC2 expression in neocortex revealed a significant decrease (*F* (4,15) = 4.868, *P* < 0.01 at 1 day; and *P* < 0.001 at 3 days) post-TBI (Fig. 1f–j). Consistent with these findings, qPCR also revealed a significant increase in NKCC1 expression and a decrease in KCC2 expression in neocortex after TBI (Supplementary Fig. 1). These results were similar to those observed in the hippocampus (Supplementary Fig. 2). Therefore, using multiple methodologies, we demonstrate elevated NKCC1 and decreased KCC2 after TBI in neocortex and archicortex. We also demonstrate that NKCC1 peaks in neocortex at 3 days post-injury (Fig. 1).

### The early post-traumatic seizures observed with behavior and EEG

**Fig. 2:**
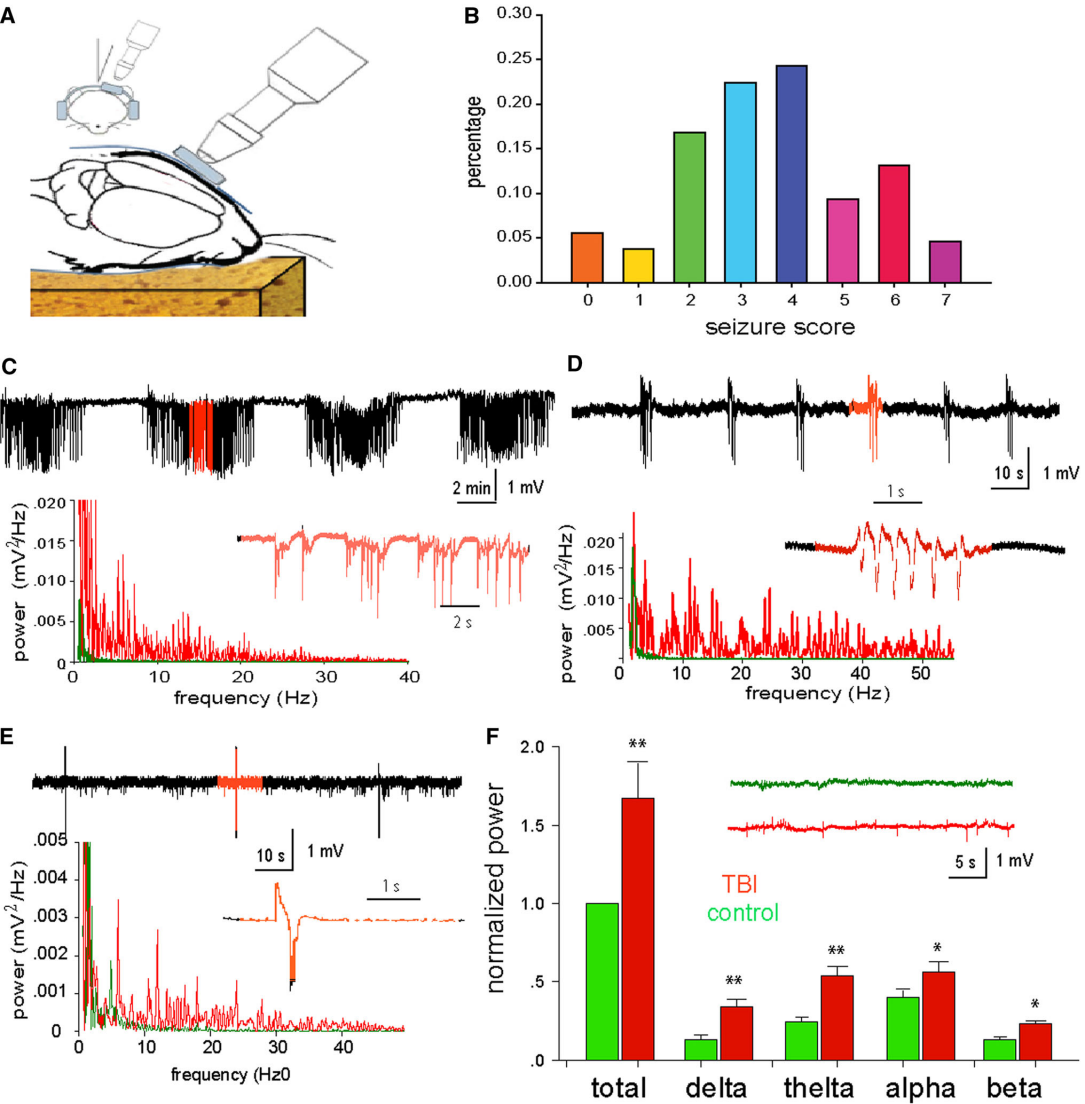
Assessment of the early post-traumatic seizures using behavioral and EEG measures. **a** A schematic diagram illustrating the TBI method is shown. **b** Analysis of the early behavioral seizures after TBI is shown to illustrate the different incidence rates of behavioral seizures using a Racine scale. **c** Extracellular field potential recording of tonic–clonic seizures in adult mice is provided. The lower panel is the power spectrum analysis of tonic (*red*) and clonic (*green*) EEG recordings. **d**–**e** Extracellular field potential recording of myclonic seizure and jerking seizures in adult mice is presented. The lower panels are the power spectrum analyses of ECoG recordings. **f** The statistical analysis of different EEG waveforms is shown from mice which did not show behavioral seizures, but did show EEG changes

We used a closed-head mouse injury model (Fig. 2a) that is adapted from the controlled cortical impact (CCI) model, as previously described (Petraglia et al. 2014a, b). Within hours after injury, the majority of the animals (73.7 %, 75 mice from total 102) exhibited behavioral manifestation of seizures (Fig. 2b), including chewing and head bobs, tonic–clonic forepaw, and hind-paw activity, and brief episodes of muscle jerks as defined with the modified Racine Scale (Medina-Ceja et al. 2012; Bergstrom et al. 2013). These episodes were typically accompanied by seizure-like EEG activity (Fig. 2c–e) based on frequency, amplitude, intensity, and waveform abnormalities (Abidin et al. 2011; Beamer et al. 2012). Interictal, tonic, and clonic discharges can also be characterized, as can periodic ictal discharges and power of EEG activity (Dzhala et al. 2005; Ferrie 2005). Of the mice (26.3 %, *N* = 27) that did not display behavioral signs of seizures, EEG recordings still showed seizure-like activity (Fig. 2f) in 21 out of the 27 mice that did not exhibit behavioral seizures. In sham-operated mice (*N* = 12), neither behavioral manifestations nor EEG signs of seizures were noted.

### NKCC1 knockout mice showed reduced early post-traumatic seizures and resistance to second-hit PTZ seizure challenge

**Fig. 3:**
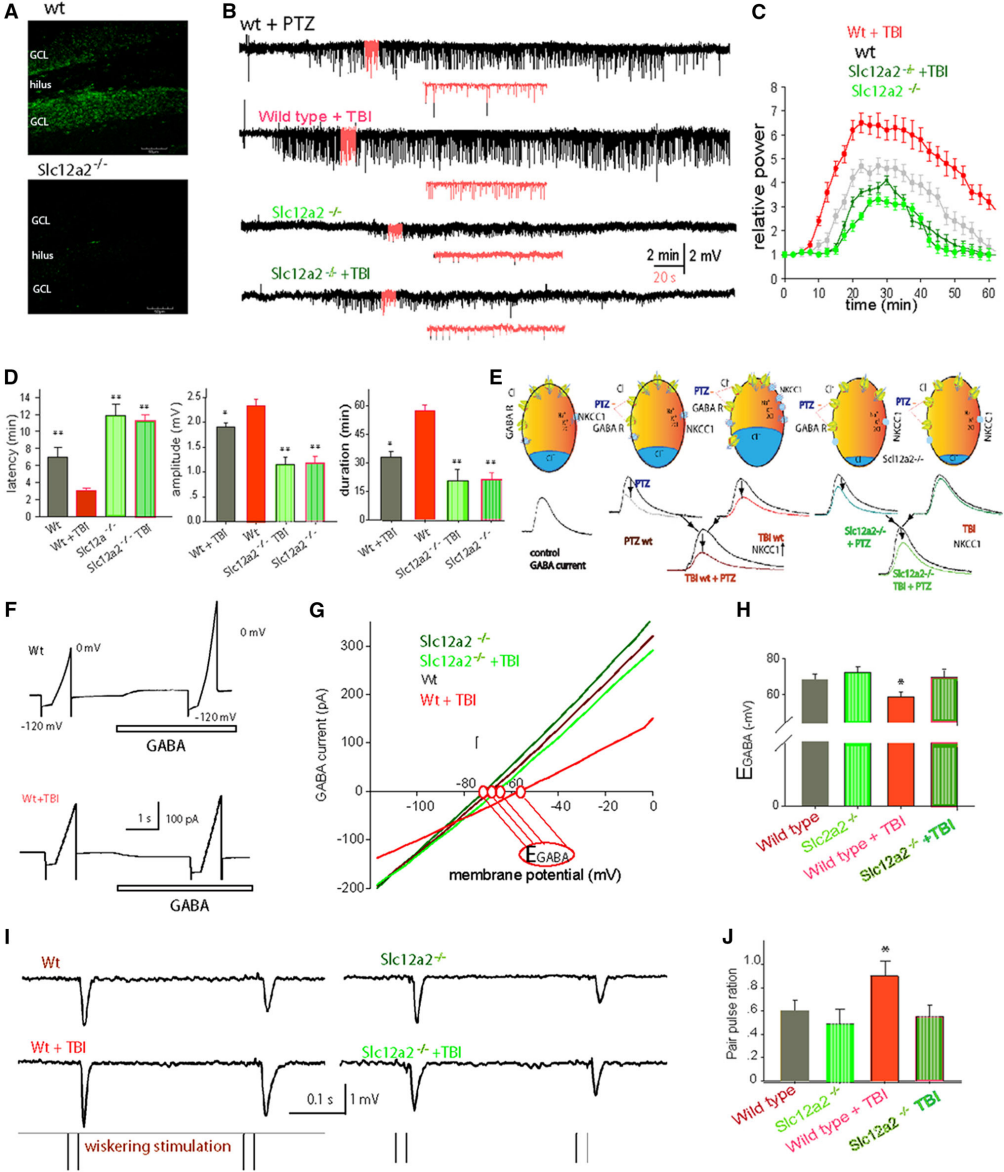
Slc12a2^−/−^ mice showed less PTZ-induced seizures and inhibited *E*_GABA_. **a** Confocal micrographs are provided to illustrate typical NKCC1 staining in the dentate gyrus (*top panel*), whereas little to no NKCC1 expression is observed in the Slc12a2^−/−^ mice (*bottom panel*). **b** Extracellular field potential recording of myoclonic seizure in adult mice induced by IP injection of PTZ (10 mg/kg). Representative traces from Slc12a2^−/−^ mice, their litter mates, with/without TBI. **c** Normalized power of ECoG after IP injection of PTZ, averaged data from five mice from each group. **d** Histograms comparing the latency, amplitude, and duration of seizures in the groups (**P < 0.01, one-way ANOVA, *N* = 5–7 animals). **e** Schematic images show that both the effects of PTZ- and TBI-induced NKCC1 up-regulation decreased the GABA currents, and blocked the effects of GAGA inhibition; therefore, PTZ can lower the threshold of NKCC1 to induce seizures. **f** Cortical neurons were clamped at −60 mV and a ramp of holding potentials was applied to induce currents before and after application of GABA (100 µM, 2 s). GABA sensitive currents–voltage (*I*–*V*) curve was plotted from ramp-induced current after GABA subtracted before. **g** Typical *I*–*V* curves show that the GABA-induced currents–voltage relationship changes after TBI in wild-type mice and in Slc12a2^−/−^ mice. The reversal potential of GABA currents (*E*_GABA_) is marked as red circles. **h** Comparisons of *E*_GABA_ in the four groups (*P < 0.01, one-way ANOVA, *N* = 5–7 slices). **i** Typical traces showed paired-pulse stimulation-induced EPSPs in vivo. **j** Comparisons of paired-pulse ratio different situations (*P < 0.01, *t* test, *N* = 5 slices)

Considering that NKCC1 is increased after TBI, we next wondered if knocking out NKCC1 would reduce the early post-traumatic seizures. To accomplish this, we used a transgenic mouse strain, in which the exon 9 of the gene-encoding NKCC1, Slc12a2 was disrupted. Immunohistochemistry for anti-NKCC1 confirmed the deletion of NKCC1 expression in the brains of the Slc12a2^−/−^ mice (Fig. 3a). Following TBI, none of the homozygous Slc12a2^−/−^ mice (*N* = 6) exhibited behavioral seizures, whereas >70 % of WT mice experienced the early post-traumatic seizures. Thus, the Slc12a2^−/−^ mice seem to be less susceptible to the early post-traumatic seizures.

To further assess the seizure susceptibility of the Slc12a2^−/−^ mice, we compared the Slc12a2^−/−^ mice with their wild-type litter mates on a PTZ (10 mg/kg, i.p.) challenge at 3 days post-injury. For these studies, we selected the six Slc12a2^−/−^ mice from above, as well as the six WT mice that neither experienced behavioral or EEG seizures. After PTZ injection, an expected pattern of behavioral seizures emerged in all of the animals that were accompanied by a 3–9 Hz polyspike EEG discharge typical of myoclonic seizures (Fig. 3b). Despite the fact that all of the animals exhibited seizure-associated behaviors, the results showed that PTZ-induced seizure severity was significantly decreased in Slc12a2^−/−^ mice compared with the wild-type mice. The average latency of PTZ-induced seizures in Slc12a2^−/−^ mice (11.2 ± 1.7 min) was significantly (*F* = 9.64 and *P* < 0.001) longer than in WT mice (3.4 ± 0.5 min). We also examined the seizure latency after a PTZ challenge in Slc12a2^−/−^ mice (12.7 ± 1.8 min) that were only exposed to a sham TBI (*N* = 6), and observed no significant differences compared with the Slc12a2^−/−^ mice that received a TBI and then PTZ challenge (Fig. 3b–d). The maximum amplitude was also significantly (*F* = 5.27 and *P* < 0.001) lower in the Slc12a2^−/−^ mice, from 2.4 ± 0.7 mV in wild type to 1.1 ± 0.2 mV in Slc12a2^−/−^ mice, and 1.2 ± 0.4 mV in Slc12a2^−/−^ mouse following TBI (Fig. 3b–d). There was no significant difference in maximum amplitude between Slc12a2^−/−^ TBI group and Slc12a2^−/−^ sham group.

### In vitro electrophysiology and in vivo electrophysiology reveal a potential NKCC1 mechanism of hyper-excitability after TBI

We assessed Cl^−^ by measuring the reversal potential of GABA_A_ currents using perforated patch on cortical slices prepared from animals at day 3 after TBI and using voltage-clamp recording from layer II cortical neurons. The results showed that *E*_GABA_ was −69.4 ± 1.9 mV in wild-type sham mice (mean ± SEM, *N* = 6 slices, Fig. 3e, f). In contrast, the reversal potential of neurons from the same layer II of wild-type TBI mice was only 58.3 ± 1.5 mV (*N* = 7 slices, *P < 0.01, one-way ANOVA). No significant differences in reversal potential were observed comparing the Slc12a2^−/−^ with wild-type littermates (*P* = 0.272, *N* = 5, Fig. 3g, h).

Most rapid synaptic inhibition in the vertebrate forebrain is mediated by GABA acting via GABA_A_ and GABA_B_ receptors (Wilcox and Dichter 1994). Here, we used the paired-pulse stimulation paradigm to test the in vivo excitability of the network in the TBI mice. Figure 3i shows a typical response for two inter-deflection intervals (200 ms). Note that, in general, there was an attenuated response to the second pulse relative to the response to the first pulse, even though the two deflections were identical in structure (Fig. 3i). However, after TBI, the paired pulse changed significantly, from 0.54 ± 0.07 to 0.88 ± 0.05 (*P < 0.01, *N* = 5, *t* test). Alternatively, in Slc12a2^−/−^ mice, the TBI did not induce any changes, from 0.46 ± 0.05 to 0.44 ± 0.03 (*P* = 0.373, *N* = 5, *t* test, Fig. 3j). These results suggest the intriguing possibility that TBI compromises cortical inhibition via an NKCC1-mediated mechanism.

### NKCC1 inhibitor bumetanide lowered seizure incidence

NKCC1 up-regulation may contribute to increased seizure susceptibility which occurs after TBI. NKCC1 is selectively blocked by low micromolar concentrations of the loop diuretic bumetanide (Isenring et al. 1998). By reducing intracellular Cl^−^ accumulation, this diuretic shifts *E*_GABA_ to negative potentials, resulting in more effective inhibition (Dzhala et al. 2005). To determine if NKCC1 protects against the early post-traumatic seizures, bumetanide (30 mg/kg, dissolved in DMSO, then diluted with 0.9 % saline) was injected 1 h before TBI. Analysis of behavioral seizures and EEG recordings following bumetanide treatment revealed a significant decrease in seizures compared with controls (41.6 %, *N* = 12 in bumetanide-treated group, as compared with 73.7 % in control, *N* = 102).

**Fig. 4:**
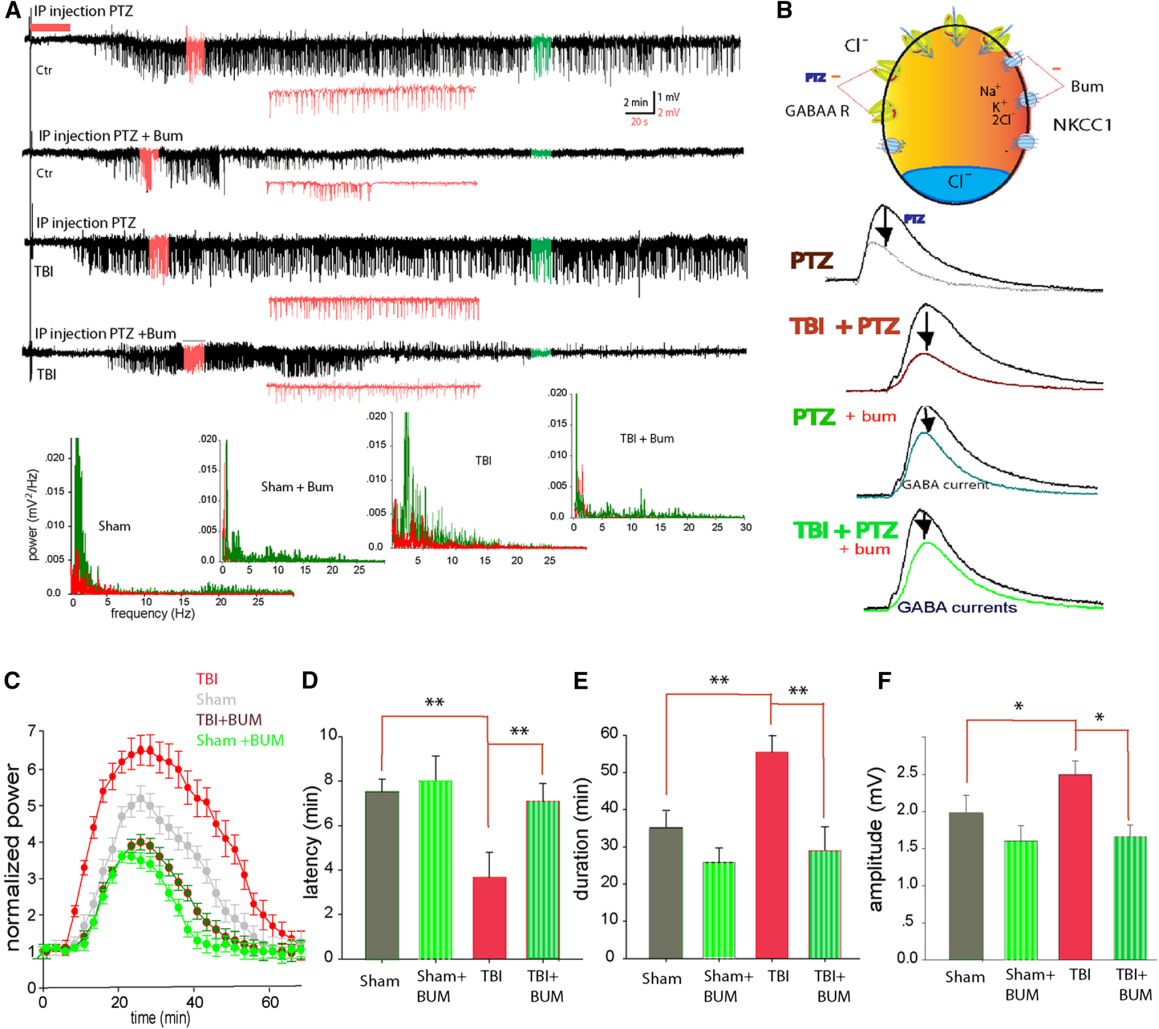
Bumetanide blocked PTZ-induced post-traumatic seizures. **a** Representative traces from wild type, wild type with TBI, and with/without i.p. injection of bumetanide 30 min before application of PTZ. *Inlets* in *red* are extension of the EEG traces. *Lower panels* are power spectra of seizure activity corresponding to the traces above, *red graph* is before, and *green graph* is after PTZ. **b** A schematic model showing the putative effects of PTZ-induced blockade of GABA receptors lowered the threshold of NKCC1 co-transporters-induced seizures, and bumetanide can reverse the effects of NKCC1-induced seizures. **c** Normalized power of EEG after i.p. injection of PTZ, averaged data from five mice from each group. The power of EEG was analyzed with power spectrum in Clampfit, and the total power at different frequency was added together to get normalized power. **d** Histogram comparing the latency of seizures in the groups (**P < 0.01, ANOVA with Newman–Keuls test, *N* = 5 animals). **e**, **f** Histograms comparing the duration and maximal amplitude (averaged top 5 %) of neuronal seizure activity detected within the 60-min observation period after application of PTZ (**P < 0.01, ANOVA with Newman–Keuls test, *N* = 5 animals)

To further determine if NKCC1 inhibition with bumetanide would alter post-traumatic seizure susceptibility, we again used the second-hit PTZ seizure challenge. After application of PTZ, the animals showed behavioral seizures and EEG recording spikes characteristic of myoclonic seizures (Fig. 4a). Statistical analysis of overall seizure activity revealed a significant difference (Fig. 4c). The latency to seizure onset was delayed in the TBI mice treated with bumetanide prior to PTZ (mean 6.98 ± 0.50 min), compared with TBI mice treated with vehicle prior to PTZ (mean 3.12 ± 0.23 min). The mean duration of the ictal episode in the bumetanide-treated group decreased significantly to 23.08 ± 1.95 and 24.8 ± 2.3 min, respectively (*F* = 13.5, *P* < 0.01, one-way ANOVA, Fig. 4d, e). The maximum amplitude of the spikes was also significantly decreased in the bumetanide group (*F* = 11.3, *P* < 0.01, one-way ANOVA, Fig. 4f). Thus, inhibiting NKCC1 with bumetanide reduced the early post-traumatic seizures and decreased susceptibility to second-hit PTZ-induced seizures.

### TGF-β: a putative factor for NKCC1 up-regulation and altered neuronal function

**Fig. 5:**
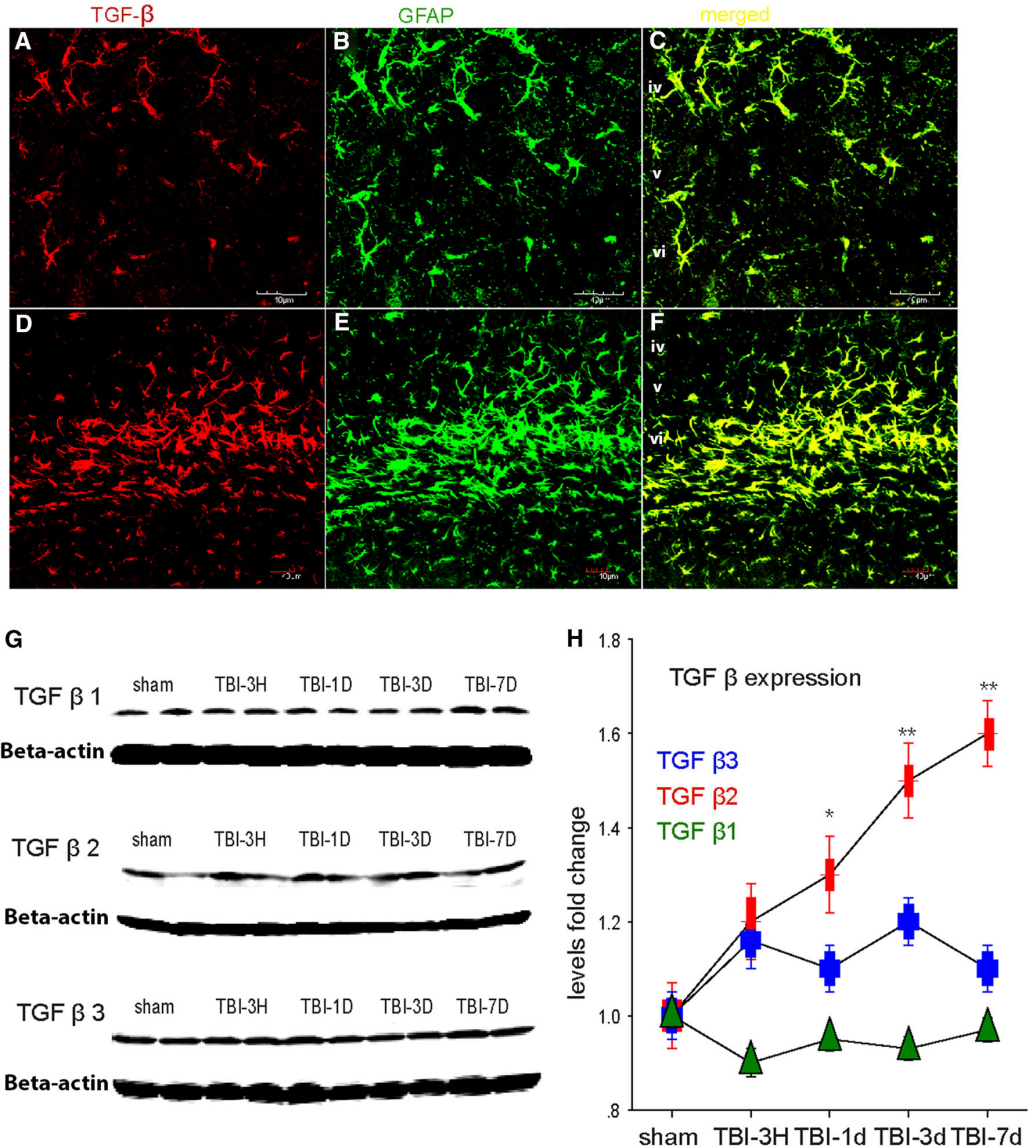
TGF-β expression observed after TBI. **a** Confocal micrographs illustrating TGF-β2-labeling in layers iv-vi of neocortex, and co-expression with GFAP-labeled astrocytes in shams (**a**–**c**) and 3 days after TBI (**d**–**f**). Note that at 3 days, after TBI, there is an increase in both TGF and GFAP-labeling. This increase may be all, or partly the result of astrocytosis after TBI. *Scale bar* 40 µm. **g** Western blot analysis confirms a significant increase in the expression of different subtypes of TGF-β in cortex. **h** Graph depicting the mean fold increase in TGF-β subtypes. (*P < 0.01, **P < 0.001, one-way ANOVA, *N* = 5)

**Fig. 6:**
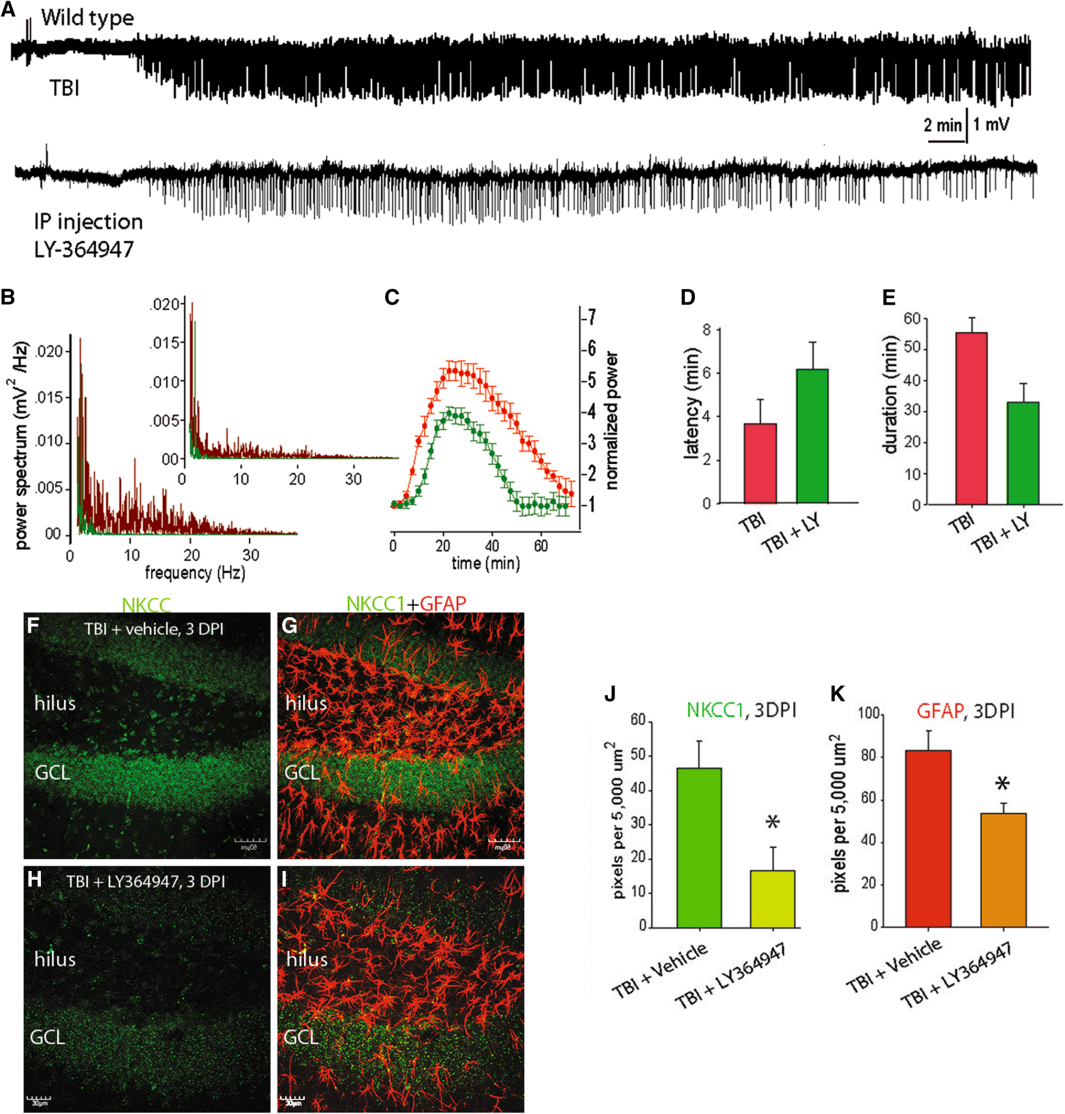
TGF-β blocker LY-364947 blocked the seizure and up-regulation of NKCC1 after TBI. **a** Representative traces of EEG recordings show that i.p. injection of TGF-β blocker LY-364947 blocked the seizures induced by TBI. **b** Power spectrum analysis of EEGs. **c** Normalized power spectrum analysis of EEG recordings. **d**, **e** Comparisons of the latency and duration of seizures induced by PTZ in addition to TBI. **f**–**i** Representative confocal micrographs of a hippocampi stained with antibody for NKCC1 (*green*; **f**, **h**) and merged with GFAP (*red*; **g**, **i**) to illustrate that that the NKCC1 expression appears to be reduced at 3 days after injury in the LY-364947-treated mice (**h**, **i**), compared with vehicle-treated mice (**f**, **g**). Note that the intensity of GFAP-labeled astrocytes also appears to be lower in the mice treated with LY-364947. These changes are most evident in the granule cell layer (GCL) and hilus, and there appears to be minimal overlap between the NKCC and GFAP staining. **j** Graph of the mean optical density of NKCC1-labeling in the hippocampal dentate gyrus at 3 days post-injury (DPI) showing significantly less staining in mice treated with LY-364947 compared with vehicle-treated mice (*P < 0.0001). **k** Graph of the mean optical density of GFAP-labeling in TBI + vehicle mice and TBI + LY-364947 mice confirmed the observation of significantly decreased GFAP-labeling, suggesting a possible decrease in astrocytosis and/or astrocyte hypertrophy following treatment (*P < 0.001). *Scale bar* **f**, **g** 50 µm; **h**, **i** 30 µm

TGF-β released from astrocytes or microglial cells has been shown to have neuroprotective effects, including improved function and decreased lesion size (Mannix and Whalen 2012; Logan et al. 2013). In the peripheral nervous system, the previous studies have shown that WNKs [with no lysine (K)], interact with TGF-β (Lee 2007) and modulate NKCC1 and KCC2 activity (Richardson and Alessi 2008). Another study concluded that the interaction between WNKs and NKCC1 might play an important role in spinal cord injury (Lee et al. 2013). Therefore, we sought to determine if TGF-β might be related to TBI-induced alterations in NKCC-1. We performed immunolabeling and western Blot, and found that TBI resulted in increased TGF-β in both cortex and hippocampus (Fig. 5a–h). To further explore the relationship of TGF-β with TBI-induced seizures and the expression of NKCC1, we used the TGF-β blocker LY-364947 (10 mg/kg, i.p., once daily for 3 days). It was found that after injection of LY364947, the animals showed less PTZ-induced seizures 3 days after TBI (Fig. 6a, b). More specifically, both the latency and duration of seizures were reduced (Fig. 6c–e). Decreased expression of NKCC1 in the brain was also observed following the LY364947 treatment (Fig. 6f–h).

## Discussion

In this study, we present data showing that TBI leads to the early post-traumatic seizures, increased NKCC1 and decreased KCC2. Although we cannot rule out the possibility that it is the seizures that cause the increased NKCC1, not the TBI, our data support a role for NKCC1 in the early post-traumatic seizures, because deletion of NKCC1 in knockout mice decreased the seizure tendency after TBI. The fact that NKCC1 inhibition also decreased the TBI-induced positive shift of *E*_GABA_, and blocked TBI-induced network excitation supports a mechanistic role for NKCC1 in post-traumatic hyper-excitability and seizures. We provide functional evidence in support of this mechanistic role showing that pharmacological inhibition of NKCC1 using bumetanide also decreased the incidence of the early post-traumatic seizures. Furthermore, antagonizing TGF-β resulted in decreased NKCC1 expression and a decrease in seizure activity following TBI and a second-hit PTZ challenge. Therefore, our study identifies NKCC1 and TGF-β as potential new targets for the treatment of TBI-induced seizures.

In this study, we were able to clearly identify a role for NKCC1 in the GABA inhibitory pathway that was down-regulated due to elevated intracellular Cl^−^ levels and a depolarized equilibrium potential. NKCC1 is selectively blocked by low micromolar concentrations of the loop diuretic bumetanide (Isenring et al. 1998). By reducing intracellular Cl^−^ accumulation, this diuretic shifts *E*_GABA_ to negative potentials, resulting in more effective inhibition (Yamada et al. 2004; Dzhala et al. 2010). We used bumetanide and Slc12a2^−/−^ mice to inhibit the elevated NKCC1 after TBI. Both of these treatments decreased seizure susceptibility following TBI, supporting a mechanistic role for elevated NKCC1 in post-traumatic seizures.

NKCC1 is an electron-neutral cotransporter that imports Na^+^, K^+^, and Cl^−^, with the net effect of raising intracellular Cl^−^ concentration, resulting in a depolarized *E*_GABA_. Raising intracellular Cl^−^ concentration might affect extracellular K^+^ buffering, which can increase seizure susceptibility (Wang et al. 2012b). Moreover, shifts in *E*_GABA_ can initiate a positive feedback cycle in neonatal seizures (Staley and Smith 2001), by reduced efficacy of GABA-enhancing anticonvulsants (Dzhala and Staley 2003; Dzhala et al. 2008), which are the first-line agents in the treatment of neonatal seizures (Rennie and Boylan 2003; Carmo and Barr 2005). Therefore, elevated NKCC1 might increase post-traumatic seizure susceptibility by direct modulation of GABA currents, or indirect modulation via changes in Cl^−^ and K^+^. Future studies are needed to decipher the precise mechanisms.

TGF-β is a pleiotropic cytokine that plays a pivotal role in intracellular communications and is involved in cell growth, embryogenesis, morphogenesis, wound healing, and immune responses. TGF-β up-regulation is involved in many disease conditions, and it has also been shown to be elevated in the cerebrospinal fluid of patients following TBI (Dohgu et al. 2005; Phillips et al. 2006). Our results demonstrate that blocking the TBI-induced increase in TGF-β results in a decrease in the expression of NKCC1 and reduced seizure activity (Fig. 6). These data are the first to demonstrate a putative link between TBI, TGF-β, NKCC1, and physiological alterations. Although many researchers consider TGF-β to be a protective cytokine (Brionne et al. 2003), others have also demonstrated that it can exacerbate excitotoxicity (Perillan et al. 2002; Mesples et al. 2005). While the potential involvement of TGF-β in seizure pathology has previously been reported, the detailed mechanism and differential cellular pathways bridging TGF-β signaling to seizures are still a matter of investigation (Heinemann et al. 2012).

## Conclusion

We report the novel and important finding that reversing or deleting the TBI-induced increase in NKCC1 resulted in a decrease in the early post-traumatic seizures and a reduction in post-traumatic seizure susceptibility. We also demonstrate several putative mechanisms through which elevated NKCC1 might be involved in increased post-traumatic seizures susceptibility. These results are consistent with the reports of a seizure-promoting role of NKCC1 in neonatal seizures and in other adult seizure disorders. Therefore, NKCC1 may be a novel therapeutic target for the treatment of TBI and related disorders, including seizures.

## Electronic supplementary material

Below is the link to the electronic supplementary material.

**Supplemental Figure S1. qPCR shows increased NKCC1 and decreased KCC2 in the neocortex after TBI.** In addition to the western Blot and quantitative immunocytochemistry, we also assessed expression levels using qPCR. The results show that in left neocortex, expression is already significantly increased (F, 4(4,25) = 1.850, P < 0.05) by 3 h after TBI. This increase is observed at least until 7 days after TBI. In the right cortex, NKCC1 expression is significantly increased (F (4,25) = 4.995, P < 0.05) at 3 and 7 days after TBI (TIFF 2219 kb)

**Supplemental Figure S2. Increased NKCC1 in the hippocampus following TBI**. Although we performed our electrophysiological recordings in cortex, we also performed cellular and molecular analysis of NKCC1 in the hippocampus. Expression of NKCC1 was significantly increased (F (4,25) = 1.361, P < 0.05) in the left hippocampus (**A**) at 3 h, 1 and 3 days after TBI, and in (**B**) the right hippocampus (F (4,25) = 3.864, P < 0.05) at 1, 3, and 7 days after TBI. We further assessed immunohistochemical labeling for NKCC1 in the hippocampus of sham (**C**), 1 day post-TBI (**D**), and 3 days post-TBI (**E**) mice. Note the increasing NKCC1 staining in the supra- and infra-pyramidal blades of the granule cell layer (GCL). Densitometric analysis (**F**) revealed that at 1 and 3 days after TBI, NKCC1 is increased in the dentate gyrus (TIFF 10349 kb)
