# Supplementary figures and images for: NKCC1 up-regulation contributes to early post-traumatic seizures and increased post-traumatic seizure susceptibility
Source: Brain Struct Funct. 2016 Sep 1;222(3):1543–56. doi: 10.1007/s00429-016-1292-z (PMC5368191; doi:10.1007/s00429-016-1292-z)

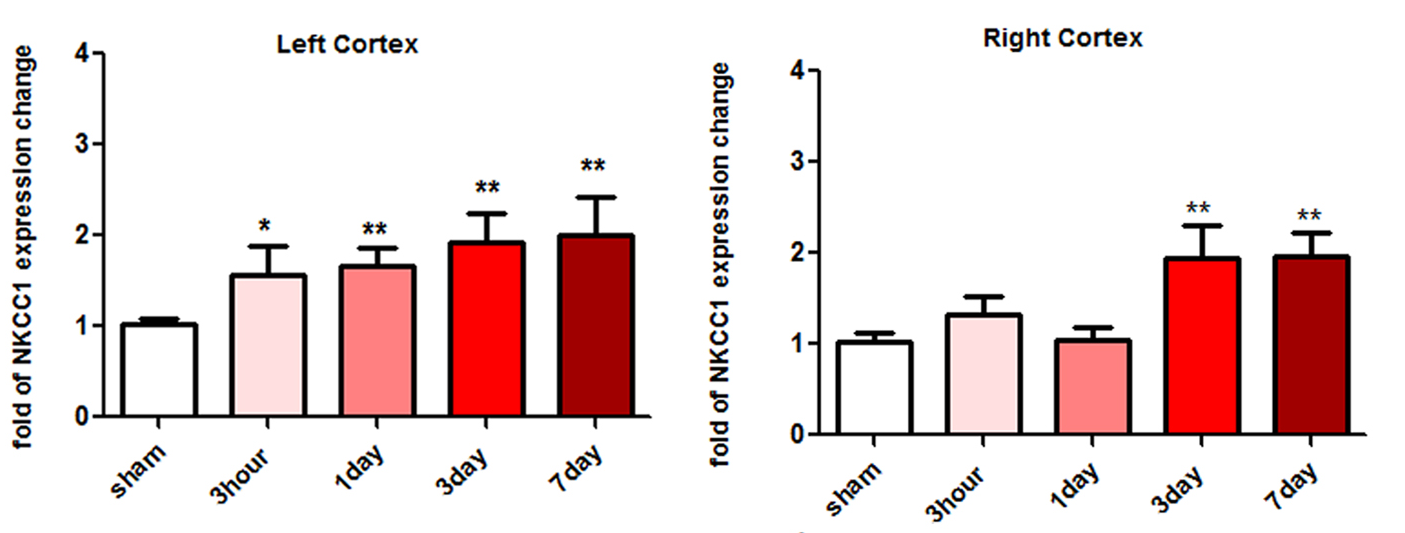

Supplement: Supplementary file 1 — Supplemental Figure S1. qPCR shows increased NKCC1 and decreased KCC2 in the neocortex after TBI. In addition to the western Blot and quantitative immunocytochemistry, we also assessed expression levels using qPCR. The results show that in left neocortex, expression is already significantly increased (F, 4(4,25) = 1.850, P < 0.05) by 3 h after TBI. This increase is observed at least until 7 days after TBI. In the right cortex, NKCC1 expression is significantly increased (F (4,25) = 4.995, P < 0.05) at 3 and 7 days after TBI (TIFF 2219 kb) [file 429_2016_1292_MOESM1_ESM.tif]

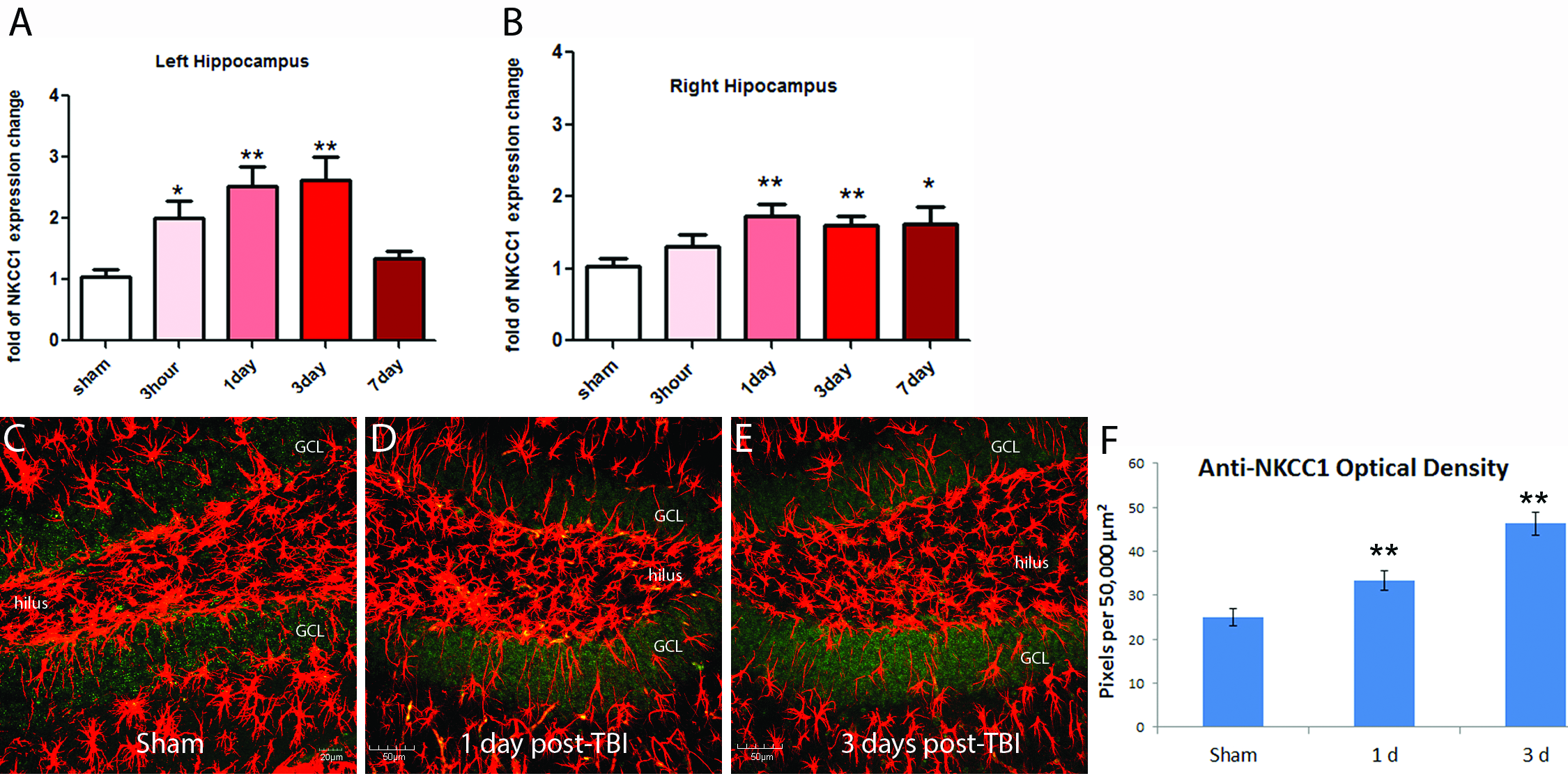

Supplement: Supplementary file 2 — Supplemental Figure S2. Increased NKCC1 in the hippocampus following TBI. Although we performed our electrophysiological recordings in cortex, we also performed cellular and molecular analysis of NKCC1 in the hippocampus. Expression of NKCC1 was significantly increased (F (4,25) = 1.361, P < 0.05) in the left hippocampus (A) at 3 h, 1 and 3 days after TBI, and in (B) the right hippocampus (F (4,25) = 3.864, P < 0.05) at 1, 3, and 7 days after TBI. We further assessed immunohistochemical labeling for NKCC1 in the hippocampus of sham (C), 1 day post-TBI (D), and 3 days post-TBI (E) mice. Note the increasing NKCC1 staining in the supra- and infra-pyramidal blades of the granule cell layer (GCL). Densitometric analysis (F) revealed that at 1 and 3 days after TBI, NKCC1 is increased in the dentate gyrus (TIFF 10349 kb) [file 429_2016_1292_MOESM2_ESM.tif]
